# Rare case of low-grade extranodal NK/T-cell lymphoma, nasal type, arising in the setting of chronic rhinosinusitis and harboring a novel N-terminal *KIT* mutation

**DOI:** 10.1186/s13000-018-0765-1

**Published:** 2018-11-23

**Authors:** Kyle Devins, Stephen J. Schuster, Gabriel C. Caponetti, Agata M. Bogusz

**Affiliations:** 1 Department of Pathology and Laboratory Medicine, Division of Hematopathology, Hospital of the University of Pennsylvania, 7.018 Gates Pavilion, 3400 Spruce Street, Philadelphia, PA 19104-4283 USA; 2 Lymphoma Program, Abramson Cancer Center, University of Pennsylvania, Philadelphia, PA 19104 USA

**Keywords:** NK/T-cell lymphoma, Epstein-Barr virus, Indolent, Low-grade, *KIT* mutation

## Abstract

**Background:**

Extranodal NK/T-cell lymphoma, nasal type (ENKTCL-NT), is a rare aggressive subtype of non-Hodgkin lymphoma characterized by angioinvasion, angiodestruction, necrosis and strong association with Epstein-Barr virus (EBV). ENKTCL-NT occurs worldwide and is more prevalent in Asian and the Native American populations of Mexico, Central and South America. It represents approximately 10% of all peripheral T-cell lymphomas worldwide. The aim of this report is to present a rare case of ENKTCL-NT with an unusually indolent clinical course and low-grade histopathologic features.

**Case presentation:**

A 71-year-old Asian woman with a long-standing history of seasonal rhinosinusitis presented with persistent nasal congestion, cough, and fever unresponsive to antihistamines and antibiotics. Histopathological evaluation of a polypoid nasal mass revealed an atypical infiltrate with predominantly small lymphoid cells that were CD2+, surface CD3-, cytoplasmic CD3+, CD5(dim)+, CD7(dim)+, cytotoxic markers (granzyme B and perforin)+, EBER+ and CD56-. The Ki-67 proliferative index was very low (< 1%). T-cell receptor gamma gene rearrangement studies were positive for a monoclonal rearrangement, and sequencing studies identified a novel *KIT* mutation (p. K167 M, c. 500 A > T). A diagnosis of low-grade ENKTCL-NT was rendered.

**Conclusions:**

Our case of ENKTCL-NT is unusual due to (1) an indolent clinical course (2) low-grade histopathologic features including a low proliferative index (3) lack of CD56 expression and (4) a novel *KIT* mutation. This case raises awareness of the existence of a subset of cases of ENKTCL-NT that can potentially be misdiagnosed as a reactive process, particularly in patients with recurrent chronic rhinosinusitis.

## Background

Extranodal NK/T-cell lymphoma, nasal type (ENKTCL-NT), is a distinct form of non-Hodgkin lymphoma (NHL) characterized by an aggressive clinical course and a strong association with Epstein-Barr virus (EBV) [1]. The disease is most common in Asians and the Native American populations of Mexico, Central America and South America, while occurrence in the United States and Europe is relatively rare [1]. The reported median age of patients ranges from 44 to 54 years, and it is more commonly seen in males [1]. Patients with nasal involvement typically present with a destructive sinonasal mass that can be accompanied by epistaxis [1]. Involvement of other body sites is less commonly observed, with cutaneous involvement being most frequent [1, 2]. EBV appears to be implicated in its pathogenesis, given that the lymphoma cells are almost invariably infected with the clonal episomal form of the virus [3–6]. The histopathologic features share an angiocentric and angiodestructive growth pattern regardless of anatomic location, with mucosal ulceration in the setting of nasal involvement [1]. The cytological spectrum of the lymphoma cells is broad and may include small to large cells, with or without anaplastic morphology. However, the majority of cases feature medium-sized cells or a mixture of small and larger cells with irregular nuclei, inconspicuous nucleoli and moderate amounts of often pale or clear cytoplasm with occasional azurophilic granules [1, 7]. The lymphoma cells are occasionally accompanied by a variety of inflammatory cells, in which case the infiltrate can mimic a reactive process. The neoplastic cells are typically positive by in situ hybridization for EBV-encoded RNA (EBER) and by immunohistochemical staining for CD2, cytoplasmic CD3ε, CD56 and cytotoxic markers such as perforin, granzyme B and TIA-1; immunohistochemical staining is usually negative for surface CD3 [1, 8]. Although a variety of cytogenetic alterations have been described, with del(6)(q21q25) being the most frequently identified, none appear to be specific [1, 9]. Recent genome-wide sequencing studies have provided an insight into the pathogenesis of ENKTCL-NT. Recurrent mutations in genes that encode proteins involved in the JAK/STAT, PI3K/AKT, NOTCH, PDGFR and KIT signaling pathways suggest that these may play a role in the pathogenesis of ENKTCL-NT and represent potential therapeutic targets [1, 9–12]. The clinical course of the ENKTCL-NT is typically highly aggressive and historically it has been associated with poor survival rates (30–40%) [1] . Although recent advances in therapy, including upfront radiotherapy, have improved the outcomes [10–12], there is still a need for more effective treatments, particularly in advanced cases [1, 12, 13].

In this report, we present a rare case of ENKTCL-NT with unusual clinicopathologic features akin to those seen in chronic rhinosinusitis and compare it with similar cases reported in the literature.

## Case presentation

### Clinical findings

A 71-year-old Asian woman presented for the evaluation of persistent nasal congestion, cough and fever. She was born in India but lived most of her life in the United States. Her past medical history was significant for seasonal allergic rhinosinusitis that was most severe in early summer. During the last recurrence, the symptoms of nasal congestion were unresponsive to antihistamines. Subsequently, she developed cough and low-grade fever and received two courses of oral prednisone and antibiotics for 6 weeks, without symptom resolution. A computer tomography (CT) scan revealed mucosal thickening in the maxillary sinuses, bilaterally. Two months after the initial onset of symptoms, functional endoscopic sinus surgery was performed and a polypoid nasal mass was removed. Upon histopathological evaluation at an outside institution, a diagnosis of ENKTCL-NT was rendered. Serological studies for EBV IgM were within normal limits, while EBV capsid IgG was elevated. Polymerase chain reaction (PCR) for EBV DNA performed on the patient’s serum yielded results below the limit of detection for the assay. The patient subsequently transferred her care to our institution, and by then she had been completely asymptomatic for several weeks. A follow-up CT scan showed persistent mucosal thickening, and a repeat nasal biopsy was performed at our institution.

### Histology and immunohistochemistry

**Fig. 1:**
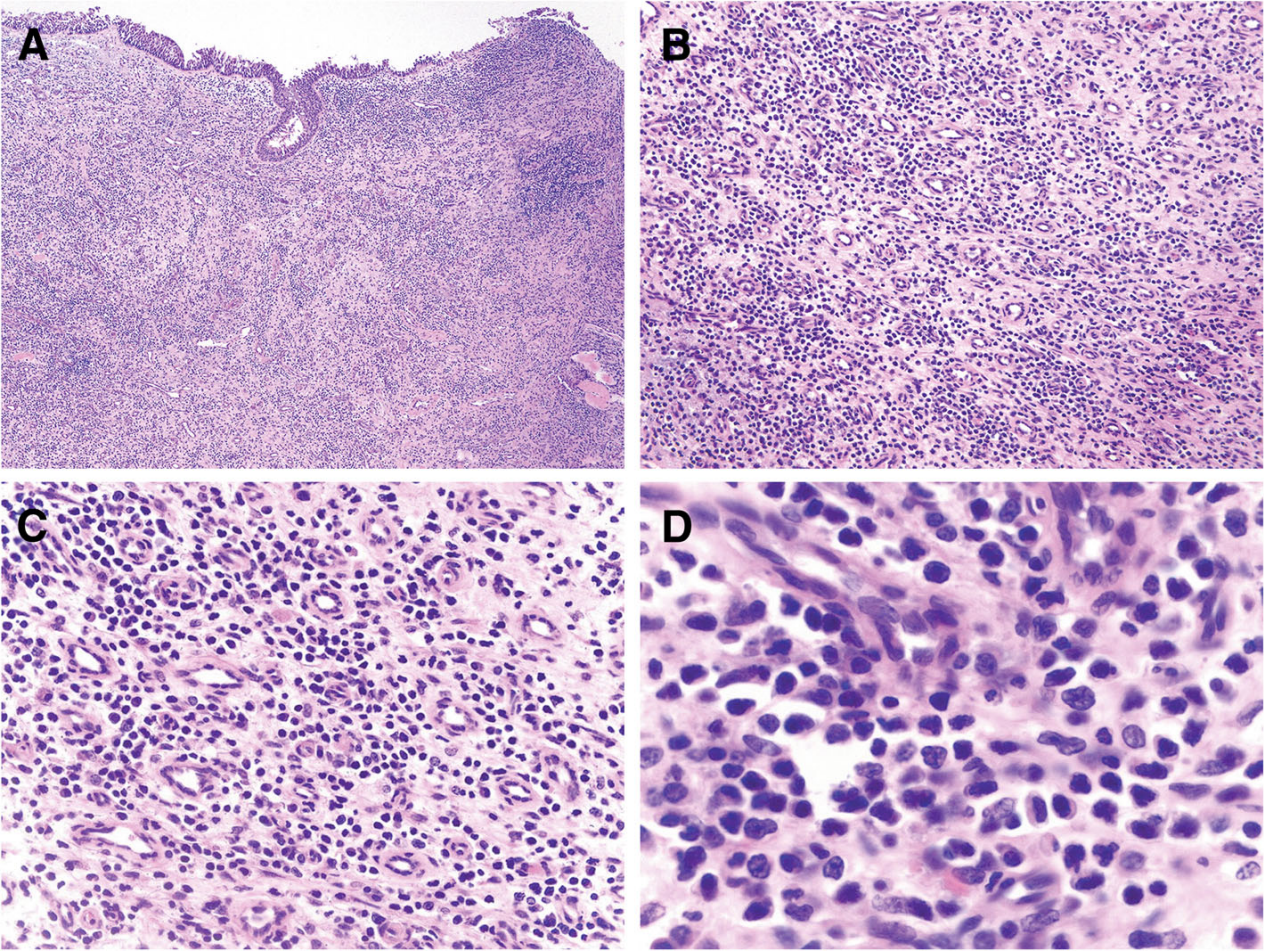
Histological findings of the nasal biopsy. **a** H&E-stained sections at low magnification (50x) demonstrate a diffuse infiltrate in the submucosa (H&E). At higher magnification **b** 200x and **c** 400x), the majority of the cells are small to medium-sized and have irregular nuclei and inconspicuous nucleoli. **d** High magnification (1000x) highlights the irregular nuclear contours

**Fig. 2:**
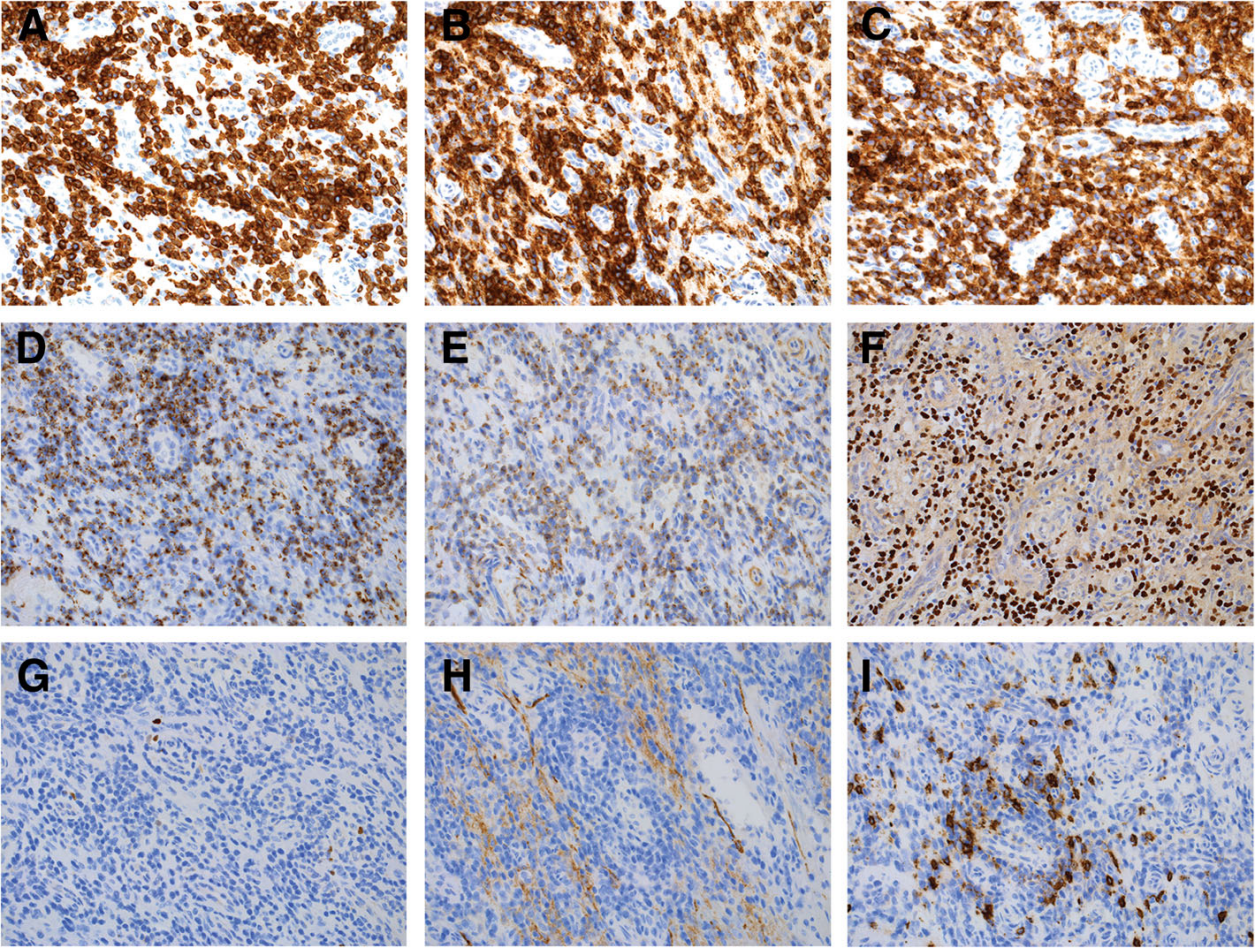
Immunohistochemical findings of the nasal biopsy. **a** CD3 (200x) **b** CD2 (200x) **c** CD5 (200x) **d** granzyme B (200x) **e** perforin (200x) **f** EBER **g** Ki-67 **h** CD56 (200x) **i** CD20 (200x)

The repeat nasal biopsy contained fragments of fibrotic sinonasal mucosa with a diffuse infiltrate (Fig. 1a and b) composed of atypical small to medium-sized lymphoid cells (Fig. 1b and c) with hyperchromatic, irregularly folded nuclei (Fig. 1d). The overlying sinonasal mucosa showed focal ulceration and although there was focal angiocentricity, no necrosis was observed. Immunohistochemical evaluation demonstrated that the atypical lymphoid cells were positive for cytoplasmic CD3 (Fig. 2a), CD2 (Fig. 2b), CD5 (Fig. 2c), granzyme B (Fig. 2d), perforin (Fig. 2e) and EBER (Fig. 2f). The Ki-67 proliferative rate was low (< 1% overall) (Fig. 2g). The atypical cells were negative for CD56 (Fig. 2h), CD20 (Fig. 2i), CD4, CD7, CD8, TIA-1, CD30 and CD57.

### Flow cytometry

**Fig. 3:**
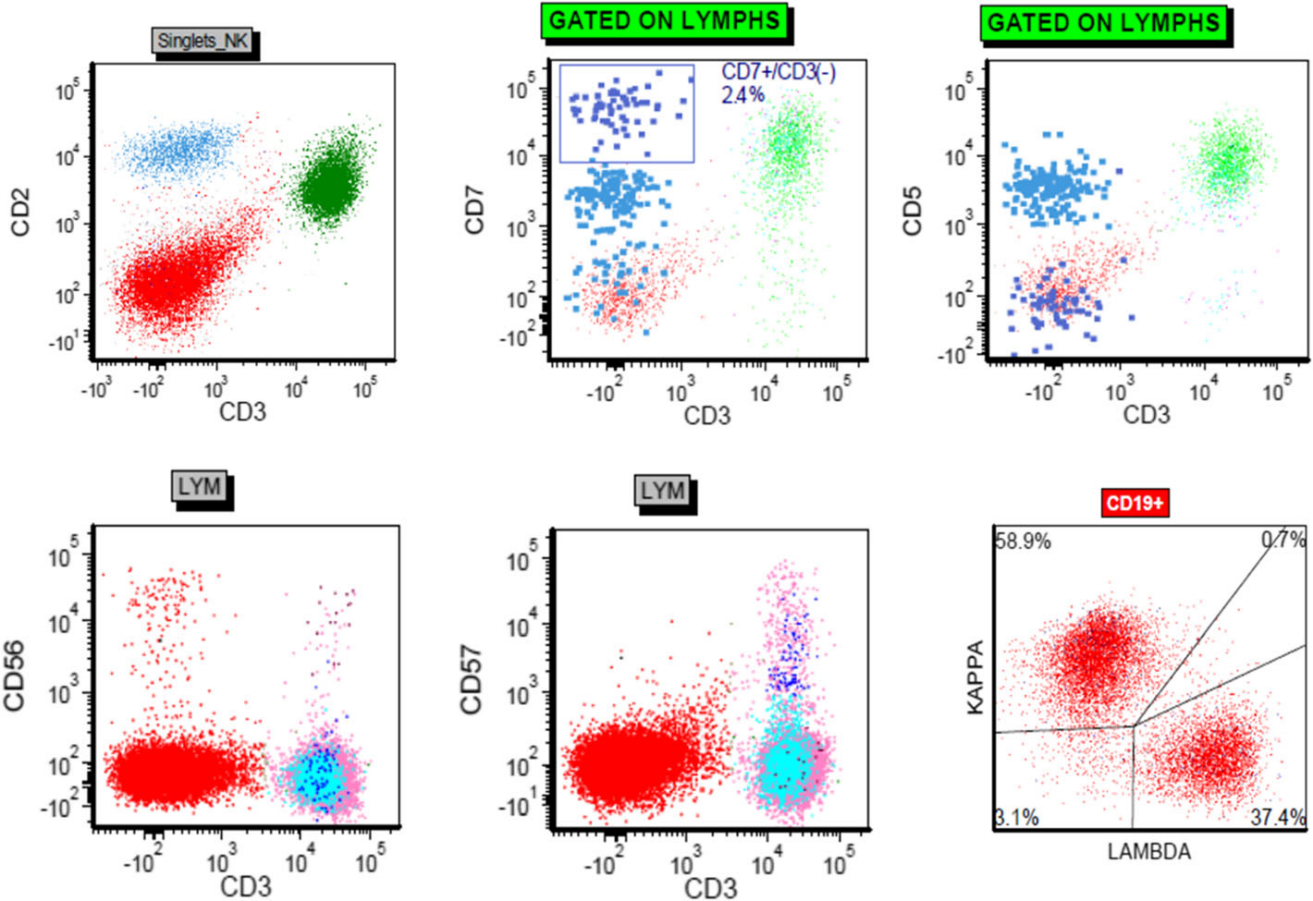
Flow cytometric analysis of representative tissue from the nasal biopsy. The aberrant lymphoid population (light blue) is surface CD3-, CD2+, CD5(dim)+, CD7(dim)+, CD56- and CD57- . Also present are normal CD3+ T cells (green) without significant loss of pan T-cell antigens (green) as well as normal CD3- and CD7+ NK cells (dark blue) and polyclonal B-cells (red)

Flow cytometric studies performed on tissue fragments from the nasal biopsy demonstrated a subset of lymphoid cells, comprising 6% of total events, with the following immunophenotype: CD2+, surface CD3-, CD4-, CD8-, CD5(dim)+, CD7(dim)+, CD16-, CD25-, CD56- and CD57- (Fig. 3). Also present were CD3+ T cells without significant loss of pan T-cell antigens and a CD4:CD8 ratio of 4:1 (53% of total events) as well as few unremarkable CD3- CD7+ NK cells and polyclonal B-cells.

### T-cell receptor rearrangement studies

**Fig. 4:**
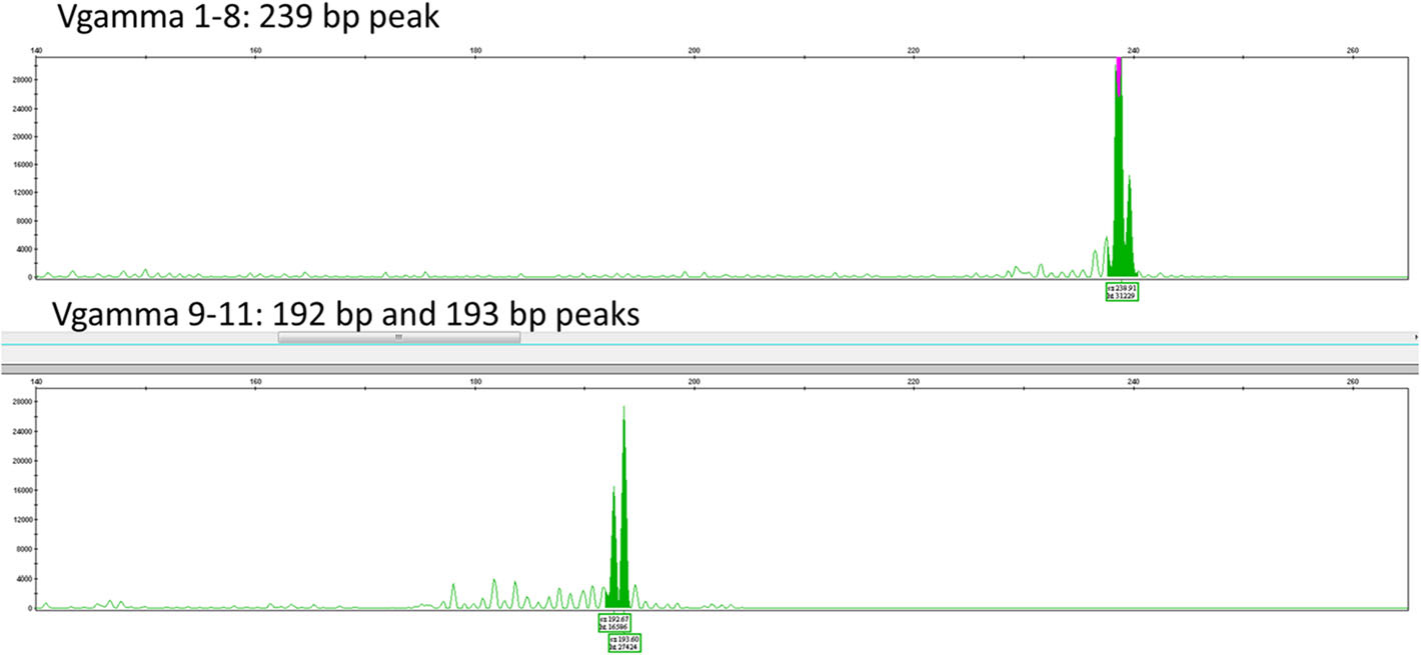
T-cell receptor (TCR) gamma gene rearrangement studies by PCR performed on the nasal biopsy. The TCR gamma gene rearrangement studies revealed a monoclonal rearrangement with a 239 base pair peak in the V-gamma Reaction 1, and 192 and 193 base pair peaks in the V-gamma Reaction 2

T-cell receptor gamma gene rearrangement studies by PCR revealed a prominent 239 base pair peak in one reaction, and 192 and 193 base pair peaks in an irregular polyclonal distribution in a second reaction (Fig. 4).

### Gene sequencing studies

Next-generation sequencing studies performed at the University of Pennsylvania using a custom, targeted sequencing amplicon panel for 68 hematologic malignancy-associated genes [14, 15] revealed a missense variant in exon 3 of the *KIT* gene at amino acid 167, converting the wild type residue lysine to methionine (p.K167 M, c.500A > T), with an allele frequency of 23%.

## Discussion

Clinically, indolent ENKTCL-NT appears to be an exceedingly rare entity as only seven other cases have been reported in the literature (Table 1). All previously reported cases demonstrated variably dense lymphocytic infiltrates without angioinvasion or necrosis [16–19]. Cytologic atypia, in the form of nuclear membrane irregularity, was present in only a few cases, and the findings often imitated reactive inflammatory infiltrates [19, 20]. Although the number of cases described in the literature is low, the reported immunophenotypic results appear to show uniform expression of CD2, cytoplasmic CD3 and granzyme B and EBER, and variable expression of CD56. Five cases involved the sinonasal tract only, while two cases initially presented in the skin. Among the sinonasal cases with available clinical histories, low-grade ENKTCL-NT developed in patients with a long-standing history of chronic rhinosinusitis, often in association with nasal polyps [17, 18]. In one report, a case of a CD20-positive ENKTCL-NT arose in a patient with a 10-year history of rhinorrhea and the lymphoma reportedly exhibited an indolent clinical course; however, this case showed aggressive morphologic features and the follow-up of 6 months was too short to confirm the clinical behavior of the neoplasm [21].

**Table 1:** Reported cases of ENKTCL with low-grade histological findings

Case	Citation	Gender	Age	Site	Presentation	Angioinvasion/ necrosis	Atypia	Ki-67	Immunophenotype	Description/Follow-up
1	Seishima et al. [16]	F	60	Skin	Recurrent swelling upper lip initially diagnosed as “lymphoid proliferation associated with EBV”	No	No	NR	CD45RO+, EBER+, CD56-, CD20-	Developed ENKTCL-NT with angioinvasion and necrosis involving facial skin and nasal tract 9.5 years later, died despite treatment
2	Zhang et al. [17]	F	53	Sinonasal	History of chronic rhinosinusitis with nasal polyps removed 20 years prior; Progressive nasal obstruction	No	Mild	80%	cCD3+, CD56+, Granzyme-B+, EBER+, TIA-1+, CD20-	Treated with chemotherapy and radiation, disease-free at 4 month follow-up
3	Tabanelli et al. [18]	M	52	Sinonasal	History of chronic rhinosinusitis for 13 years with eventual sinonasal thickening obliterating right maxillary sinus and nasal turbinate	No	Mild	Moderately high	CD2+, CD3+, CD56+, Granzyme-B+, EBER+, CD4-, CD5-, CD7-, CD8-	Initially diagnosed as EBV-associated proliferation, persisted for 2 years and diagnosed as ENKTCL-NT on a rebiopsy; long term follow-up not available
4–6	Hasserjian et al. [19]	NR	NR	Sinonasal	unknown	No	No	NR	NR	Remained indolent for 10 years, recurred secondary to immunosuppression for renal transplant
		NR	NR	Sinonasal	unknown	No	No	NR	NR	Bland histologic appearance resembling inflammatory infiltrate, no further description
		NR	NR	Sinonasal	unknown	No	No	NR	NR	
7	Zuriel et al. [20]	F	55	Skin	Large red violaceous infiltrated plaques	No	No	> 90%	CD2+,cCD3+, CD56-, Granzyme B+, EBER+, CD4-, CD5-,CD8-	Indolent course over 22 years with only local skin recurrences treated by local radiotherapy
8	Current Case	F	71	Sinonasal	History of longstanding chronic rhinosinusitis, sinonasal thickening/polyp	No	Mild	< 1%	CD2+, cCD3+, CD5(dim)+, Perforin+, Granzyme-B+, EBER+, CD56-, CD30-	Treated with radiation, no recurrence at 6 month follow-up

*cCD3* Cytoplasmic CD3, *NR* Not reported

The Ki-67 proliferation index in our case was unusually low (< 1%) and this is, to the best of our knowledge, the lowest percentage of Ki67+ cells reported in ENKTCL-NT. Low Ki-67 expression (defined as positive in < 65% of the lymphoma cells) has been shown to be predictive of a better prognosis in patients with stage I/II ENKTCL-NT [22]. The absence of CD56 expression seen in our case is another unusual feature in ENKTCL-NT with sinonasal location, although this was also seen in the two previously reported indolent cases with cutaneous involvement (Table 1) [16, 20].

Treatment with chemotherapy and/or radiation was administered in several of the reported cases, with a favorable clinical course after short-term follow-up, and one case demonstrated spontaneous remission without therapy [23]. The two indolent cases of ENKTCL-NT involving the skin were initially diagnosed as reactive inflammatory processes. [16, 20] While one of the latter cases had an indolent course over 22 years with only local skin recurrences [20], the other case recurred at 9.5 years with both nasal and cutaneous involvement, angioinvasion, necrosis, and an aggressive clinical course despite treatment [16]. In the report by Tabanelli et al [18], the authors document a case of low-grade ENKTCL-NT that may have progressed from a polymorphic EBV-related NK/T-cell proliferation.

Although EBV appears to play a role in the development of ENKTCL-NT, the molecular pathogenesis of the disease has not been yet fully elucidated. Recurrent mutations, deletions and hypermethylation have been described in several genes, including *KMT2D*, *ARID1A, TP53, KIT, JAK3*, *STAT3*, *STAT5B,CTNNB1, BCOR*, *ASXL3*, *EP300*, *DDX3X*, *ECSIT, KRAS*, *NRAS, PRDM1, BCL2L11* (*BIM*), *DAPK1*, *PTPN6* (*SHP1*), *TET2*, *SOCS6*, and *ASNS* [24, 25]. Interestingly, our case showed a previously unreported mutation in exon 3 of *KIT* with an allele frequency of 23%, which strongly suggests that this is a somatic mutation. The proto-oncogene *KIT* encodes a receptor tyrosine kinase that plays an important role in many cellular processes including hematopoiesis, and therapeutic targeting of KIT signaling in cancer is an area of intense research [26]. Mutations of *KIT* are associated with several human malignancies, most notably gastrointestinal stromal tumors, acute myeloid leukemia, mastocytosis and melanoma. To date, little is known about the role of *KIT* mutations in the pathogenesis of ENKTCL-NT. Exon 11 or exon 17 *KIT* mutations have been identified in 10 to 71.4% of ENKTCL-NT cases from Asia [27]. The reported exon 11 and exon 17 mutations comprise “hot spots” within the extracellular juxtamembrane domain and the activation loop of the kinase domain, respectively. However, a gain of function as a result of these KIT mutations has not been demonstrated [27]. The exon 3 mutation seen in our case (p. K167 M, c. 500 A > T) is located within the second Ig-like N-terminal domain of KIT, which is involved in ligand binding [25]. Identification of this novel *KIT* mutation might contribute to elucidating the pathogenesis of the indolent cases of ENKTCL-NT.

## Conclusions

In summary, we present a unique case of indolent ENKTCL-NT with low-grade histopathologic features (including an extremely low proliferation index), unusual lack of CD56 expression, and a novel *KIT* mutation.

Our findings raise awareness of the existence of this apparently rare but likely underreported entity which can potentially be mistaken for a reactive process, and underlines the need for a thorough morphologic, immunohistochemical and molecular evaluation for its diagnosis.

## Abbreviations

CT: Computer tomography
EBER: EBV-encoded RNA
EBV: Epstein-Barr virus
ENKTCL: Extranodal NK/T-cell lymphoma
ENKTCL-NT: Extranodal NK/T-cell lymphoma, nasal type
NHL: Non-Hodgkin lymphoma
PCR: Polymerase chain reaction
